# Serum and Urinary Magnesium Status in Asian CKD Patients and Healthy Controls: A Cross-Sectional Analysis

**DOI:** 10.3390/nu18101542

**Published:** 2026-05-13

**Authors:** Umer Farooq Khan, Chelsea Wei Ling Chia, Gek Cher Chan, Boon Wee Teo

**Affiliations:** 1 Division of Nephrology, Department of Medicine, National University Hospital, Singapore 119074, Singapore; chelsea.chia@mohh.com.sg (C.W.L.C.); gek_cher_chan@nuhs.edu.sg (G.C.C.); mdctbw@nus.edu.sg (B.W.T.); 2 Division of Nephrology, Department of Medicine, Yong Loo Lin School of Medicine, National University of Singapore, Singapore 119074, Singapore

**Keywords:** magnesium, deficiency, CKD, urinary

## Abstract

Background: Magnesium is an essential dietary mineral, and low magnesium status has been associated with adverse cardiometabolic and renal outcomes. In chronic kidney disease (CKD), the prevalence of magnesium deficiency remains uncertain because serum magnesium alone may not accurately reflect overall magnesium status. We aimed to characterize magnesium status in a multi-ethnic Asian CKD cohort compared with healthy participants using combined serum and 24-h urinary magnesium (24U-Mg) measurements. Methods: This cross-sectional observational study included 232 adults with CKD and 103 healthy participants. Serum magnesium and 24-h urinary magnesium excretion were measured concurrently. Magnesium deficiency was defined as serum magnesium ≤0.75 mmol/L; probable magnesium deficiency was defined as serum magnesium 0.76–0.85 mmol/L with 24U-Mg ≤ 3.29 mmol/day; and possible magnesium deficiency was defined as either normal serum Mg with low 24U-Mg ≤ 1.65 mmol/day or serum Mg 0.76–0.85 mmol/L with 24U-Mg > 3.29 mmol/day. Associations with age, sex, body mass index, diabetes, blood pressure, and kidney function were examined. Results: CKD participants had lower mean serum magnesium (0.86 vs. 0.90 mmol/L, *p* < 0.001) and lower 24U-Mg (2.50 vs. 2.93 mmol/day, *p* = 0.006) compared with healthy participants. Using the proposed combined serum and 24U-Mg criteria, magnesium deficiency was present in 13.8% of CKD participants and 0% of healthy participants, while probable deficiency was observed in an additional 25.8% of CKD and 16.5% of healthy participants. Multivariate analysis demonstrated that CKD, older age, high BMI, and diabetes status were independently associated with lower serum magnesium levels, and female sex was associated with lower serum and urinary magnesium in healthy participants. Conclusions: Magnesium deficiency is common in non-dialysis CKD patients and is frequently not identified by serum magnesium alone. Combined assessment using serum and urinary magnesium may better identify individuals at risk of magnesium deficiency and inform future prospective studies in CKD.

## 1. Introduction

Magnesium is an essential cation involved in numerous biochemical processes, including neuromuscular function, bone health, glucose metabolism, and cardiovascular homeostasis [1,2]. As a dietary mineral, magnesium plays a critical role in maintaining metabolic and cardiovascular health across the lifespan. The kidneys play a pivotal role in magnesium balance by adjusting reabsorption and excretion in response to bodily needs. Therefore, chronic kidney disease (CKD) can disrupt magnesium homeostasis in complex ways. Reduced glomerular filtration in advanced CKD may lead to hypermagnesemia due to impaired excretion. However, many CKD patients have concurrent risk factors for magnesium deficiency such as poor dietary intake, diabetes mellitus, diuretic therapy, and gastrointestinal losses which may offset the tendency for magnesium retention [3]. Low magnesium status is a concern in CKD because hypomagnesemia has been associated with a higher risk of cardiovascular events, vascular calcification, and all-cause mortality [1]. Yet despite its potential significance, the true prevalence of magnesium deficiency in CKD remains uncertain, partly due to challenges in assessing magnesium nutritional status.

Serum magnesium is the most utilized measure of magnesium status, but it is an unreliable indicator of total body magnesium [4,5]. Over 99% of magnesium resides in tissues and bone, with less than 1% foundin blood. Serum concentrations are tightly regulated and may not decline until significant depletion has occurred in tissues. Indeed, individuals with “normal” serum magnesium (conventional reference range of 0.70–1.10 mmol/L) can still have subclinical magnesium deficits. For instance, some experts consider serum Mg in the range of 0.75–0.85 mmol/L as “chronic latent Mg deficiency”, or CLMD, which may be clinically relevant despite falling within the “normal” range [4,6]. Reliance on serum magnesium alone may therefore underestimate the burden of magnesium insufficiency, especially in CKD patients who often have only mild hypomagnesemia.

A more comprehensive approach is to complement serum measurements with 24-h urinary magnesium excretion (24U-Mg) to gauge magnesium status [7]. Twenty-four-hour urinary magnesium excretion is increasingly recognized as a functional biomarker of magnesium intake and balance, reflecting the integrated effects of intestinal absorption and renal conservation. In magnesium deficiency, the kidneys typically conserve magnesium, resulting in low 24-h excretion. Conversely, an inappropriately high urinary Mg excretion in the context of low serum Mg suggests renal magnesium wasting [5]. Controlled metabolic studies have shown that individuals consuming inadequate magnesium (<250 mg/day) have a characteristically low urinary magnesium range (40–80 mg/day or 1.65–3.29 mmol/day), while those with magnesium intake >250mg/day have a higher range (80–160 mg/day, or 3.30–6.60 mmol/day) independent of sex [2,4,7]. Thus, a 24U-Mg level below 3.30 mmol per day may indicate insufficient dietary intake or depleted magnesium stores. Combining thresholds of low serum Mg (≤0.85 mmol/L) with low 24U-Mg (≤3.29 mmol/day) may potentially identify individuals who are truly magnesium-deficient [4,6]. This combined biochemical approach has been proposed to better characterize magnesium status than serum measures alone.

Magnesium status may also be influenced by demographic and clinical factors. The results of population-based dietary surveys suggest that magnesium deficiency is common in Asian populations. In South Korea, only ~57% of young adults met the estimated average intake requirement for magnesium [8], while in Taiwan, 12–24% had serum Mg < 0.80 mmol/L, and 74–81% of adults had suboptimal magnesium intake according to dietary recall [9]. Women, in particular, appear more prone to hypomagnesemia, with the authors of a Canadian study noting that low serum Mg was more frequent in South Asian and white women, and correlated with obesity and insulin resistance [10]. Older individuals may also be at risk due to lower dietary intake and impaired absorption. Similarly, patients with malabsorption and gastrointestinal comorbidities may also be at risk of magnesium deficiency.

In CKD, advanced age often coincides with reduced nutrient intake, and deficiency is often exacerbated by decreased calcitriol activation in renal disease reducing intestinal absorption [11]. In diabetic patients, hyperglycemia can induce urinary magnesium losses [11,12], and insulin resistance can cause magnesium to shift to the intracellular compartment, contributing to hypomagnesemia [10]. However, whether these factors translate to net deficiency in CKD patients is unclear. Diuretic medications, especially loop and thiazide diuretics, as well as immunosuppressants used to treat glomerulopathies, enhance urinary magnesium excretion and can lead to magnesium wasting over time. Proton-pump inhibitors and other medications can also impair magnesium balance by reducing gastrointestinal absorption or causing renal losses [3,13].

Despite growing recognition of the role of magnesium in CKD outcomes and the limitations of serum Mg measurement, few studies have systematically evaluated magnesium status in CKD using combined serum and urinary criteria. In this study, our objective is to investigate magnesium status in a multi-ethnic Asian cohort of CKD patients and healthy controls by using both serum magnesium and 24-h urinary magnesium excretion. We aim to (1) compare magnesium deficiency prevalence between CKD and non-CKD individuals, and (2) explore the associations of these magnesium measures with age, sex, BMI, diabetes status, and kidney function. We hypothesize that CKD patients would have a higher subclinical magnesium deficiency prevalence than healthy individuals, and that certain subgroups would be more likely to be magnesium deficient. By providing a descriptive assessment of magnesium status, we seek to identify groups at potential nutritional risk and to inform future research in CKD.

## 2. Materials and Methods

### 2.1. Study Design and Participants

A total of 335 participants (232 CKD patients and 103 controls) were included in this cross-sectional observational study which included a retrospective secondary analysis of prospectively collected data. The study size was based on the two original prospective studies’ recruitment protocol to determine a GFR regression line with stratified recruitment for ethnicity, sex, and GFR. Anonymized data was obtained from these two prior studies approved by the Doman Specific Review Board [14,15]. Healthy controls had no known kidney disease, and no major chronic illnesses or long-term medication. All participants provided written informed consent.

### 2.2. Data Collection

Demographic information (age, sex, self-reported ethnicity) and clinical data (medical history of diabetes mellitus, hypertension, and medications including diuretics) were recorded. Body weight and height were measured to calculate body mass index (BMI), and blood samples, early-morning spot urine samples, and 24-h urine collections were acquired for relevant biochemical indices. Glomerular filtration rate (GFR, mL/min per 1.73 m^2^) was measured by plasma disappearance using technetium-diethylenetriaminepentaacetic acid (Tc^99m^ DTPA).

### 2.3. Definitions of Magnesium Status

We categorized magnesium status using combined serum and 24U-Mg thresholds informed by prior research [6].

(1) Magnesium deficiency was defined as serum Mg ≤ 0.75 mmol/L (overt hypomagnesemia).
(2) Probable magnesium deficiency was defined as serum Mg 0.76–0.85 mmol/L, a range described in the literature as chronic latent magnesium deficiency [6], combined with urinary indices of 24U-Mg ≤ 3.29 mmol/day.
(3) Possible magnesium deficiency was defined as serum Mg 0.76–0.85 mmol/L (CLMD), but with higher urinary indices of 24U-Mg > 3.29 mmol/day, or replete serum Mg > 0.85 mmol/L with low 24U-Mg ≤ 1.65 mmol/day, values which likely suggest renal conservation to maintain serum Mg levels.
(4) Likely replete magnesium was defined as serum Mg > 0.85 mmol/L with 24U-Mg > 1.65 mmol/day.

### 2.4. Statistical Analysis

Participant characteristics were summarized as mean ± standard deviation for normally distributed variables or counts (percentages) for categorical variables. All participants had complete data and were included in the analysis. Continuous variables were assessed visually for distributional characteristics and analyzed on their original scale, as transformations did not improve normality. Group comparisons were made using the independent *t*-test for continuous variables (or Mann–Whitney *U* test for non-parametric data), Chi-square test for proportions, and multivariate linear regression. Relationships between magnesium indices and other variables were evaluated using Pearson correlation coefficients, as appropriate. A two-sided *p* value < 0.05 was considered statistically significant. The statistical analyses were performed using SPSS (Version 23).

## 3. Results

### 3.1. Cohort Characteristics

A total of 232 participants with chronic kidney disease (CKD) and 103 healthy participants were included in this analysis, with their baseline characteristics summarized in Table 1. Compared with healthy participants, the CKD cohort was significantly older (58.4 ± 12.8 vs. 42.5 ± 14.3 years, *p* < 0.001) and had a higher mean body mass index (27.6 ± 5.5 vs. 24.9 ± 4.0 kg/m^2^, *p* < 0.001). Diabetes and hypertension were common in the CKD group (51.3% and 82.8%, respectively), and 52.6% of CKD participants were receiving diuretic therapy. Healthy participants had no major comorbidities or long-term medication use.

### 3.2. Magnesium in CKD vs. Healthy Controls

**Figure 1:**
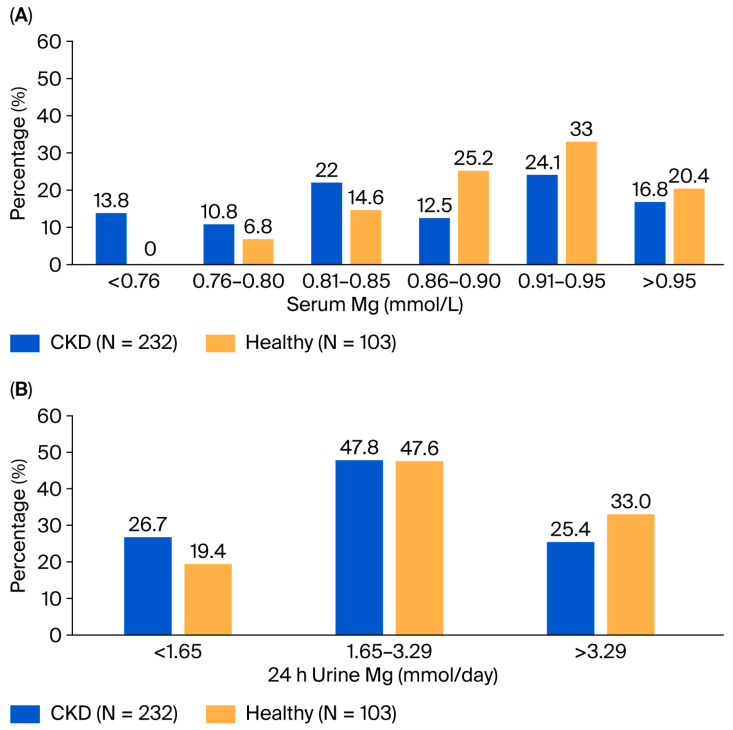
Distribution of serum Mg (mmol/L) and 24hr urinary Mg (mmol/day), among healthy and CKD participants. (**A**) distribution of serum Mg in CKD and healthy participants; (**B**) distribution of 24-h urine Mg in CKD and healthy participants. In CKD, a leftward shift in serum Mg and greater proportion of participants with 24U-Mg ≤1.65 mmol/day are noted.

Serum magnesium concentration was significantly lower in the CKD group compared to healthy controls (mean 0.86 vs. 0.90 mmol/L, *p* < 0.001). Although the absolute difference was modest (0.04 mmol/L), the distribution differed substantially between groups (Figure 1A). None of the healthy participants had serum magnesium ≤ 0.75 mmol/L, and 21.4% had ≤0.85 mmol/L. In contrast, 13.8% of CKD participants had serum magnesium ≤ 0.75 mmol/L, and 46.6% had ≤0.85 mmol/L.

Urinary magnesium excretion over 24 h (24U-Mg) was also lower in CKD participants (2.50 ± 1.25 vs. 2.93 ± 1.45 mmol/day, *p* = 0.006). As shown in Figure 1B, a greater proportion of CKD participants had 24U-Mg ≤ 1.65 mmol/day (26.7% vs. 19.4%), while a greater proportion of healthy participants had 24U-Mg > 3.29 mmol/day (33.0% vs. 25.4%).

### 3.3. Prevalence of Magnesium Deficiency by Combined Criteria

By integrating serum and urine magnesium measurements, we identified subsets of participants based on their risk of magnesium deficiency (Table 2). A total of 32 of 232 (13.8%) CKD participants had hypomagnesemia with serum Mg ≤ 0.75 mmol/L regardless of 24U-Mglevels; however, none of the healthy participants had serum Mg ≤ 0.75 mmol/L. Probable magnesium deficiency was present in 60 (25.8%) of CKD participants compared with 17 (16.5%) healthy participants, and possible magnesium deficiency was noted in 57 (26%) of the CKD participants and 26 (22.3%) of the healthy participants.

### 3.4. Association of Magnesium Indices with Patient Factors

Associations between magnesium indices and selected clinical variables in CKD participants are shown in Table 3.

**Figure 2:**
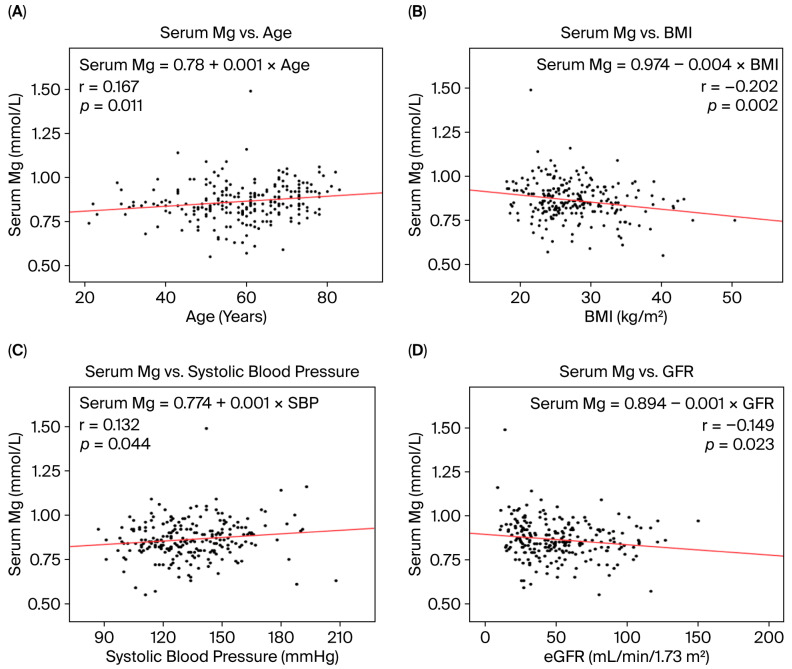
Pearson’s correlation and linear regression equations for serum magnesium (mmol/L) and (**A**) age, (**B**) BMI, (**C**) systolic blood pressure and (**D**) GFR measured using Tc^99m^ DTPA among participants with chronic kidney disease (N = 232).

Age: CKD participants with 24U-Mg ≤ 1.65 mmol/day were older than those with higher urinary magnesium excretion (62.7 ± 12.4 vs. 56.9 ± 12.6 years, *p* = 0.002). Serum magnesium showed a weak positive correlation with age among CKD participants (Figure 2A), associations that were not seen in healthy controls.

Sex: A greater proportion of female than male CKD participants had serum Mg ≤ 0.75 mmol/L (17.9% vs. 10.0%), although this difference was not statistically significant (*p* = 0.090). A similar non-significant trend was observed for 24U-Mg ≤ 1.65 mmol/day (32.1% vs. 21.7%, *p* = 0.077).

In contrast, among healthy participants, female sex was associated with lower serum magnesium < 0.85 mmol/L (36.5% female vs. 13.7% male *p* = 0.012) and lower 24-h urinary magnesium excretion ≤ 1.65 mmol/day (28.8% female vs 9.8% *p* = 0.023). Healthy males had higher average serum Mg (0.92 ± 0.06 mmol/L) than females (0.89 ± 0.07 mmol/L *p* = 0.020) and had a higher 24U-Mg levels (3.3 +/− 1.45 mmol/day) than females (2.54 +/− 1.35 mmol/day, *p* = 0.004).

Body Mass Index: Serum magnesium was inversely correlated with BMI (Figure 2B). CKD participants with BMI > 30 were more likely to have serum Mg ≤ 0.75 mmol/L, whereas urinary magnesium excretion did not differ by BMI category.

Diabetes Mellitus: Among CKD participants, diabetes was associated with a higher proportion of serum Mg ≤ 0.75 mmol/L (21.0% vs. 6.2% in non-diabetics, *p* = 0.001), but there were no significant differences in 24U-Mg levels between diabetic and non-diabetic participants.

Hypertension: Serum magnesium showed a weak positive correlation with systolic blood pressure (Figure 2C), but there were no differences in the prevalence of hypertension across serum or urinary magnesium thresholds.

Kidney Function (GFR): Estimated glomerular filtration rate (GFR) was inversely correlated with serum magnesium (Figure 2D). Participants with advanced CKD (stages G4–G5) were less likely to have 24U-Mg ≤ 1.65 mmol/day compared with those with CKD stages G1–G3 (21.1% vs. 42.6%, *p* = 0.002), while serum magnesium concentrations did not differ significantly by CKD stage.

Diuretic use: CKD participants on loop diuretics tended to have lower GFR but higher serum magnesium (0.907 ± 0.118) and FEMg (12.05% ± 8.89) compared to participants on thiazides or not on diuretics (Table 4). There were no differences in 24U-Mg. 

### 3.5. Multivariate Analysis

Multivariable linear regression analysis with both serum Mg and 24U-Mg was performed to account for the contributions of CKD stage, age, sex, BMI, and diabetes status (Table 5). The results showed a significant independent association of age, CKD stage, BMI, and diabetes status with serum Mg, and age and sex with 24U-Mg.

## 4. Discussion

### 4.1. Magnesium and CKD

In this cross-sectional study of a multi-ethnic Asian cohort, we demonstrate that magnesium deficiency is common in non-dialysis CKD and cannot be reliably identified by serum magnesium alone. Using combined serum and 24-h urinary magnesium indices, approximately 40% of CKD participants met the criteria for either overt or probable magnesium deficiency. These prevalences were substantially higher than those observed in healthy participants, despite many CKD patients having serum magnesium values within the conventional reference range.

The lower mean serum magnesium observed in CKD, together with the marked leftward shift in serum magnesium distribution, is consistent with prior observational studies reporting a high prevalence of low-normal or mildly reduced serum magnesium in CKD populations [16]. While the absolute difference in mean serum magnesium between CKD and healthy participants was small, nearly half of CKD participants had serum magnesium ≤ 0.85 mmol/L, a range increasingly recognised as biologically relevant and associated with adverse outcomes [2].

The concurrent reduction in 24-h urinary magnesium excretion among CKD participants suggests that low serum magnesium in this population is not solely attributable to renal magnesium wasting. Instead, low urinary excretion likely reflects a combination of reduced dietary magnesium intake, enhanced renal conservation, and altered magnesium handling in CKD. Using our findings, we attempt to extend prior observations of inadequate magnesium intake leading to low urinary magnesium excretion in healthy populations [4] to a CKD population. However, urinary magnesium studies should be interpreted with caution in CKD, as apart from reduced oral intake, reduced filtered load from low GFR may also reduce total 24U-Mg levels. Similarly, diuretic use may increase 24U-Mg levels and falsely provide reassurance of magnesium status. At the individual level, 24U-Mg should be interpreted in the context of dietary assessment and medications. Fractional excretion of magnesium (FEMg) measurement may also help add information on the renal handling of magnesium to better contextualize 24U-Mg levels. The underlying etiology of CKD may further influence magnesium, particularly in diabetes and hypertension, which predominated in our cohort, although the impact of less common etiologies could not be examined in detail due to limited sample sizes.

In our cohort, CKD participants receiving loop diuretics had higher mean serum magnesium levels and higher FEMg compared to those receiving thiazides, despite no significant differences in 24-h urinary magnesium excretion. Notably, participants receiving loop diuretics also had substantially lower mean eGFR, reflecting more advanced CKD. This pattern suggests that although loop diuretics increase fractional magnesium excretion through inhibition of magnesium reabsorption in the thick ascending limb of the loop of Henle, total 24-h urinary magnesium excretion may remain unchanged due to reduced filtered magnesium load in advanced CKD. These findings highlight the limitations of serum magnesium and isolated urinary magnesium measurements as markers of total body magnesium balance in CKD, where compensatory mechanisms and reduced glomerular filtration may mask underlying renal magnesium losses. In this context, FEMg may provide additional insight into renal tubular magnesium handling. Further stratified analyses by CKD stage and diuretic subtype were not performed due to limited subgroup sizes, which would reduce interpretability.

### 4.2. Age, Sex, Diabetes, and Body Composition

Older age was associated with lower urinary magnesium excretion among CKD participants, suggesting increased vulnerability to magnesium deficiency with ageing, potentially due to the contributions of age-related reductions in dietary intake, intestinal absorption, and renal adaptive capacity. Reduced calcitriol synthesis in CKD may further impair magnesium absorption, compounding age-related risk [11,17]. Female participants in the healthy controls exhibited lower serum and urinary magnesium, with similar findings having been reported in population-based studies, where women consistently demonstrated lower magnesium intake and lower serum magnesium compared with men [18]. Hormonal influences, lower dietary intake, and differences in body composition may all play a role.

We also observed an inverse association between body mass index and serum magnesium, consistent with prior studies linking obesity and insulin resistance to lower circulating magnesium concentrations [19]. This association could arise from dietary patterns low in magnesium-rich foods, and intracellular magnesium shifts in insulin-resistant states.

Diabetes was associated with a higher prevalence of low serum magnesium but not with lower 24-h urinary magnesium excretion, suggesting that, while diabetes may contribute to reduced circulating magnesium through insulin-mediated intracellular shifts or osmotic effects, it does not necessarily result in increased absolute urinary magnesium loss in established CKD. Reduced glomerular filtration may attenuate the magnesium-wasting effect of hyperglycaemia seen in populations with preserved kidney function, with similar patterns reported in CKD cohorts in studies examining magnesium handling across glycemic controls [12]. The increasing use of sodium-glucose transporter 2 (SGLT2) inhibitors in CKD populations, driven by their cardiovascular and renal protective effects, represent an important consideration for magnesium homeostasis. SGLT2 inhibitors have been associated with modest increases in magnesium in both diabetic and CKD populations independent of glucose lowering effect, possibly through altered tubular handling, although exact mechanisms are not well understood [20,21,22]. 

### 4.3. Limitations

The strengths of this study include the concurrent assessment of both serum and 24-h urinary magnesium, enabling a more nuanced assessment of magnesium status than serum measures alone. The inclusion of a healthy comparator group and the multi-ethnic Asian cohort addresses an important gap in the literature, where data on magnesium status in CKD from Asian populations is scarce.

Several limitations should be acknowledged. The cross-sectional design precludes causal inference. Second, this study is a secondary analysis of data from two prospective cohort studies; consequently, the study was not specifically designed or powered for magnesium deficiency prevalence estimates, and subgroup analysis could not be conducted. While stratified recruitment ensured the adequate representation of GFR strata and ethnic groups, it also means that the study findings are not representative of the general population but are a comparison of healthy and CKD cohorts. Due to differences in dietary practices, our study lacks applicability outside the Asian context. Third, the magnesium deficiency thresholds applied in this study are not externally validated in CKD populations or against gold-standard measures such as the magnesium loading test, and the clinical significance of “probable” and “possible” magnesium deficiency defined in this study are not well established. Lastly, the CKD cohort had a higher BMI and was older than the healthy control group which serves as a reference comparator rather than matched controls. Although multivariate linear regression was conducted to analytically address this issue, residual confounders from unmeasured factors cannot be excluded.

### 4.4. Clinical Implications

Our findings have several potential clinical implications. First, magnesium deficiency appears to be common in individuals with non-dialysis CKD and is frequently not apparent from serum magnesium measurements alone. Second, from a nutrition-focussed perspective, combined assessment using serum and 24-h urinary magnesium may help identify individuals at higher risk of magnesium deficiency, particularly older adults, women, and those with obesity or diabetes. While routine 24-h urine testing is not universally performed, it is already commonly used in nephrology practice for other indications and may provide additional value as part of a targeted nutritional assessment strategy. In more advanced CKD, 24-h urinary magnesium should be interpreted with caution, given the reduced filtration of magnesium and contextualized with a patient’s medications and nutritional assessment, and fractional excretion of magnesium may provide further insight on tubular handling of magnesium.

Emerging evidence links low magnesium status in CKD to vascular calcification, cardiovascular events, and mortality [16]. Prior interventional studies examining magnesium supplementation have shown inconsistent results, which may partially be due to heterogeneity in patient selection and use of serum Mg alone [23]. Given that serum Mg alone may not reflect magnesium depletion, future studies may consider using a combined assessment of serum and urinary magnesium to better select for patients who may benefit from magnesium supplementation. 

## 5. Conclusions

Magnesium deficiency is common in non-dialysis CKD and is frequently not detected by serum magnesium alone. Using combined serum and 24-h urinary magnesium criteria, approximately 40% of CKD participants met the criteria for overt or probably magnesium deficiency, a substantially higher prevalence than healthy participants. Older age, higher BMI, and diabetes were independently associated with lower serum Mg in CKD. Combined serum and 24-h urinary magnesium may better characterize magnesium status and inform targeted nutritional assessment strategies in nephrology practice. Prospective studies incorporating dietary assessment are needed to clarify the clinical significance and optimal management of magnesium insufficiency in CKD.

## Figures and Tables

**Table 1:** Baseline characteristics in CKD and healthy participants.

Variable	CKD (N = 232)	Healthy (N = 103)	*p*-Value
Age in years (mean, SD)	58.4 (12.76)	42.5 (14.26)	<0.001
BMI kg/m^2^ (mean, SD)	27.6 (5.45)	24.9 (4.03)	<0.001 *
Male sex (N, %)	120 (51.7)	51 (49.5)	0.709
Ethnicity (N, %)			
Chinese	94 (40.5)	35 (34.0)	<0.001
Malay	56 (24.1)	23 (22.3)	
Indian	74 (31.9)	25 (24.3)	
Other	8 (3.4)	20 (19.4)	
Diabetes (N, %)	119 (51.3)	-	<0.001
Hypertension (N, %)	192 (82.8)	-	<0.001
CKD stage			<0.001
Stage G1–G2 (GFR > 60)	72 (31.0)	-	
Stage G3 (GFR 30–60)	99 (42.7)	-	
Stage G4–G5 (GFR < 30)	61 (26.3)	-	
Cause of CKD (N, %)			
Hypertension	115 (49.6)	-	
Diabetic nephropathy	54 (23.3)	-	
Glomerular disease	38 (16.4)	-	
Other ^†^	25 (10.8)	-	
Diuretic use (N,%)			<0.001
Loop diuretic	48 (20.7)	-	
Potassium-sparing	4 (1.7)	-	
Thiazide diuretic	70 (30.2)	-	
Analytes (mean, SD)			
Serum Mg (mmol/L)	0.86 (0.11)	0.90 (0.07)	<0.001 *
Serum creatinine (mg/dL)	1.73 (1.04)	1.04 (0.19)	<0.001 *
Spot urine Mg (mmol/L)	1.63 (1.00)	1.94 (1.52)	0.059 *
24U-Mg (mmol/day)	2.50 (1.25)	2.93 (1.45)	0.006 *
Fractional excretion of Mg (FEMg)	7.36 (6.06)	3.05 (2.03)	<0.001

* Independent *t*-test: adjusted for unequal variances. ^†^ Other includes polycystic kidney disease (n = 6), obstructive disease (n = 4), and unknown (n = 15). SD = standard deviation. CKD = chronic kidney disease; GFR = glomerular filtration rate (mL/min per 1.73 m^2^) measured using Tc^99m^ DTPA; Mg = magnesium; 24U-Mg = 24 h urinary magnesium excretion. All data are presented by frequency, %, mean and standard deviation.

**Table 2:** Magnesium status of CKD and healthy participants by serum and 24-h urinary magnesium.

	CKD (N = 232)			Healthy (N = 103)		
	24U-Mg (mmol/day)					
Serum Mg (mmol/L)	≤1.65	1.66–3.29	>3.29	≤1.65	1.66–3.29	>3.29
≤0.75	13 (5.6)	14 (6.0)	05 (2.2)	00 (0.0)	00 (0.0)	00 (0.0)
0.76–0.85	17 (7.3)	43 (18.5)	25 (10.8)	06 (5.8)	11 (10.7)	9 (08.7)
>0.85	32 (13.8)	54 (23.3)	29 (12.5)	14 (13.6)	38 (36.9)	25 (24.3)

CKD = chronic kidney disease. Mg = magnesium. 24U-Mg = 24 h urinary magnesium. Total CKD participants (N = 232) and healthy participants (N = 103). All data are presented as N, and percentage of total CKD or healthy cohort participants. ■ Severe deficiency. ■ Probable deficiency. ■ Possible deficiency. ■ Likely replete.

**Table 3:** Comparison of CKD participant’s clinical characteristics across lowest threshold of serum and 24-h urinary magnesium levels.

Variables (Mean, SD)	Serum Magnesium (mmol/L)			24-Hour Urine Magnesium (mmol/day)		
	≤0.75 N = 32	>0.75 N = 200	*p*-Value *	≤1.65 N = 62	>1.65 N = 170	*p*-Value *
Age (years)	56.1 (10.9)	58.8 (13.0)	0.261	62.7 (12.4)	56.9 (12.6)	0.002
BMI (kg/m^2^)	30.3 (07.2)	27.2 (05.0)	0.025 ^‡^	27.3 (05.4)	27.7 (05.5)	0.648
Systolic BP (mmHg)	135.0 (25.6)	133.7 (20.8)	0.746	140.0 (24.7)	131.6 (19.7)	0.017 ^‡^
GFR (mL/min/1.73 m^2^)	55.0 (26.4)	51.1 (27.7)	0.468	42.0 (23.3)	55.2 (28.1)	0.001
**Variables (N, %)**	**Serum Magnesium (mmol/L)**			**24-Hour Urine Magnesium (mmol/day)**		
	**≤0.75** **N = 32**	**>0.75** **N = 200**	***p*-Value**	**≤1.65** **N = 62**	**>1.65** **N = 170**	***p*-Value ^†^**
Sex						
Male	12 (10.0)	108 (90.0)	0.090	26 (21.7)	94 (78.3)	0.077
Female	20 (17.9)	92 (82.1)		36 (32.1)	76 (67.9)	
Diabetes status						
Non-diabetic	7 (6.2)	106 (93.8)	0.001	26 (23.0)	87 (77.0)	0.237
Diabetic	25 (21.0)	94 (79.0)		36 (30.3)	83 (69.7)	
Hypertension status						
Normotensive	5 (12.5)	35 (87.5)	1.00	7 (17.5)	33 (82.5)	0.172
Hypertensive	27 (14.1)	165 (85.9)		55 (28.6)	137 (71.4)	
CKD stage						
CKD G1–G3	6 (9.8)	55 (90.2)	0.389	26 (42.6)	35 (57.4)	0.002
CKD G4–G5	26 (15.2)	145 (84.8)		36 (21.1)	135 (78.9)	
Obesity status						
BMI < 30	17 (10.1)	152 (89.9)	0.010	47 (27.8)	122 (72.2)	0.618
BMI > 30	15 (23.8)	48 (76.2)		15 (23.8)	48 (76.2)	
Diuretics						
No loop diuretics	31 (16.9)	152 (83.1)	0.005	44 (24.0)	139 (76.0)	0.140
Loop diuretics	1 (2.1%)	47 (97.9%)		17 (35.4)	31 (64.6)	
No thiazide diuretics	14 (8.7%)	147 (91.3%)	0.001	41 (25.5%)	120 (74.5%)	0.629
Thiazide diuretics	18 (25.7%)	52 (74.3%)		20 (28.6%)	50 (71.4%)	

* Independent t-test; ^†^ χ2 test; ^‡^ t-test adjusted for unequal variances. Continuous variables are expressed as mean (SD), and categorical variables as N (%). CKD = chronic kidney disease. BMI = body mass index. BP = blood pressure. GFR = glomerular filtration rate measured using Tc^99m^ DTPA.

**Table 4:** Comparison of serum and urinary magnesium indices among CKD patients taking diuretics.

	No Diuretics (N = 116)	Loop Diuretics (N = 42)	Thiazide Diuretics (N = 67)	*p*-Value *
GFR (mL/min/1.73m^2^)	61.1 ± 29.5 ^a^	27.1 ± 12.4 ^b^	51.0 ± 19.8 ^c^	<0.001
Serum Mg (mmol/L)	0.864 ± 0.098 ^ab^	0.907 ± 0.118 ^a^	0.832 ± 0.107 ^b^	0.001
FEMg (%)	6.47 ± 5.08 ^a^	12.05 ± 8.89 ^b^	6.28 ± 4.6 ^a^	<0.001
24-h Urinary Mg	2.56 ± 1.27	2.31 ± 1.18	2.61 ± 1.26	0.447

* One-way ANOVA, Values expressed in mean ± SD. ^a,b,c^: Groups sharing the same superscript letter are not significantly different on post-hoc testing. Groups with no shared letter are significantly different. GFR = glomerular filtration rate, measured using Tc^99m^ DTPA. Mg = magnesium, FEMg = fractional excretion of magnesium. Patients on potassium-sparing diuretics (N = 4), and those on multiple diuretics excluded from analysis.

**Table 5:** Multiple linear regression analysis of serum and 24 h urinary magnesium adjusted for patient characteristics (N = 335).

Variable	B (95% CI)	β	*p*-Value
Serum Mg (adjusted R^2^ = 0.086)			
CKD stage	0.040 (0.005–0.075)	0.185	0.030
Age	0.001 (0.000–0.002)	0.206	0.001
Sex	−0.004 (−0.024–0.016)	−0.022	0.671
BMI	−0.003 (−0.005–0.001)	−0.171	0.002
Diabetes status	−0.016 (−0.030–0.002)	−0.157	0.013
Hypertension	0.007 (−0.026–0.040)	0.035	0.690
24U-Mg (adjusted R^2^ = 0.104)			
CKD stage	0.214 (−0.258–0.686)	0.075	0.375
Age	−0.013 (−0.023–0.002)	−0.144	0.021
Sex	−0.726 (−0.996–−0.456)	−0.274	<0.001
BMI	0.025 (−0.002–0.052)	0.098	0.070
Diabetes status	−0.074 (−0.243–0.095)	−0.054	0.390
Hypertension status	−0.025 (−0.476–0.426)	−0.009	0.915

## Data Availability

The raw data supporting the conclusions of this article will be made available by the authors upon request.

## Abbreviations

The following abbreviations are used in this manuscript:

CKD	Chronic kidney disease
Mg	Magnesium
24U-Mg	24-h urinary magnesium
BMI	Body mass index
GFR	Glomerular filtration rate
Tc^99m^ DTPA	Technetium-diethylenetriaminepentaacetic acid

## References

[1] Kanbay M., Yilmaz M.I., Apetrii M., Saglam M., Yaman H., Unal H.U., Gok M., Caglar K., Oguz Y., Yenicesu M. (2012). Relationship between serum magnesium levels and cardiovascular events in chronic kidney disease patients. Am. J. Nephrol..

[2] Costello R.B., Elin R.J., Rosanoff A., Wallace T.C., Guerrero-Romero F., Hruby A., Lutsey P.L., Nielsen F.H., Rodriguez-Moran M., Song Y. (2016). Perspective: The Case for an Evidence-Based Reference Interval for Serum Magnesium: The Time Has Come. Adv. Nutr..

[3] Kanbay M., Goldsmith D., Uyar M.E., Turgut F., Covic A. (2010). Magnesium in chronic kidney disease: Challenges and opportunities. Blood Purif..

[4] Costello R.B., Nielsen F. (2017). Interpreting magnesium status to enhance clinical care: Key indicators. Curr. Opin. Clin. Nutr. Metab. Care.

[5] DiNicolantonio J.J., O’Keefe J.H., Wilson W. (2018). Subclinical magnesium deficiency: A principal driver of cardiovascular disease and a public health crisis. Open Heart.

[6] Rosanoff A., West C., Elin R.J., Micke O., Baniasadi S., Barbagallo M., Campbell E., Cheng F.C., Costello R.B., Gamboa-Gomez C. (2022). Recommendation on an updated standardization of serum magnesium reference ranges. Eur. J. Nutr..

[7] Costello R.B., Rosanoff A. (2020). Magnesium. Present Knowledge in Nutrition.

[8] Shim J.S., Kim K.N., Lee J.S., Yoon M.O., Lee H.S. (2023). Magnesium intake and dietary sources among Koreans: Findings from the Korea National Health and Nutrition Examination Survey 2016–2019. Nutr. Res. Pract..

[9] Wang J.L., Weng Y.L., Pan W.H., Kao M.D. (2011). Trends and Nutritional Status for Magnesium in Taiwan from NAHSIT 1993 to 2008. Asia Pac. J. Clin. Nutr..

[10] Bertinato J., Xiao C.W., Ratnayake W.M.N., Fernandez L., Lavergne C., Wood C., Swist E. (2015). Lower serum magnesium concentration is associated with diabetes, insulin resistance, and obesity in South Asian and white Canadian women but not men. Food Nutr. Res..

[11] Gröber U. (2019). Magnesium and Drugs. Int. J. Mol. Sci..

[12] Hsiao P.J., Liao C.Y., Kao Y.H., Chan J.S., Lin Y.F., Chuu C.P., Chen J.S. (2020). Comparison of fractional excretion of electrolytes in patients at different stages of chronic kidney disease: A cross-sectional study. Medicine.

[13] Pham P.-T.T., Danovitch G.M., Pham S.V. (2010). Medical Management of the Kidney Transplant Recipient. Comprehensive Clinical Nephrology.

[14] Teo B.W., Xu H., Wang D., Li J., Sinha A.K., Shuter B., Sethi S., Lee E.J.C. (2011). GFR estimating equations in a multiethnic Asian population. Am. J. Kidney Dis..

[15] Teo B.W., Xu H., Koh Y.Y., Li J., Subramanian S., Sinha A.K., Shuter B., Toh Q.C., Sethi S., Lee E.J.C. (2014). Glomerular filtration rates in healthy Asians without kidney disease. Nephrology.

[16] Sakaguchi Y., Iwatani H., Hamano T., Tomida K., Kawabata H., Kusunoki Y., Fujiyama S., Matsui I., Hayashi T., Tsubakihara Y. (2015). Magnesium modifies the association between serum phosphate and the risk of progression to end-stage kidney disease in patients with non-diabetic chronic kidney disease. Kidney Int..

[17] Rude R.K., Singer F.R., Gruber H.E. (2009). Skeletal and hormonal effects of magnesium deficiency. J. Am. Coll. Nutr..

[18] Hruby A., Guasch-Ferré M., Bhupathiraju S.N., Manson J.E., Willett W.C., McKeown N.M., Hu F.B. (2017). Magnesium Intake, Quality of Carbohydrates, and Risk of Type 2 Diabetes: Results From Three U.S. Cohorts. Diabetes Care.

[19] Guerrero-Romero F., Morales-Gurrola G., Preza-Rodríguez L., Gómez-Barrientos A., Olivas-Martínez A.I., Simental-Mendía L.E. (2022). Magnesium intake is associated with the metabolically healthy obese phenotype. J. Investig. Med..

[20] Kitao T., Konishi E., Kubori M., Komoda Y., Mori Y., Ibata T. (2025). Verification of the Short-term Effect of SGLT2 Inhibitors on Serum Magnesium Stratified by the Classification of Albuminuria Severity in Patients with Type 2 Diabetes Mellitus. Intern Med..

[21] Tang H., Zhang X., Zhang J., Li Y., Del Gobbo L.C., Zhai S., Song Y. (2016). Elevated serum magnesium associated with SGLT2 inhibitor use in type 2 diabetes patients: A meta-analysis of randomised controlled trials. Diabetologia.

[22] Shah C.V., Sparks M.A., Lee C.-T. (2024). Sodium/Glucose Cotransporter 2 Inhibitors and Magnesium Homeostasis: A Review. Am. J. Kidney Dis..

[23] Bressendorff I., Hansen D., Schou M., Silver B., Pasch A., Bouchelouche P., Pedersen L., Rasmussen L.M., Brandi L. (2017). Oral Magnesium Supplementation in Chronic Kidney Disease Stages 3 and 4: Efficacy, Safety, and Effect on Serum Calcification Propensity-A Prospective Randomized Double-Blinded Placebo-Controlled Clinical Trial. Kidney Int. Rep..

